# Early Response of Soil Properties and Function to Riparian Rainforest Restoration

**DOI:** 10.1371/journal.pone.0104198

**Published:** 2014-08-12

**Authors:** Rose Gageler, Mark Bonner, Gunnar Kirchhof, Mark Amos, Nicole Robinson, Susanne Schmidt, Luke P. Shoo

**Affiliations:** 1 School of Agriculture and Food Sciences, The University of Queensland, St Lucia, Australia; 2 Lake Baroon Catchment Care Group, Maleny, Australia; 3 School of Biological Sciences, The University of Queensland, St Lucia, Australia; USDA-ARS, United States of America

## Abstract

Reforestation of riparian zones is increasingly practiced in many regions for purposes of biodiversity conservation, bank stabilisation, and improvement in water quality. This is in spite of the actual benefits of reforestation for recovering underlying soil properties and function remaining poorly understood. Here we compare remnant riparian rainforest, pasture and reforestation plantings aged 2–20 years in an Australian subtropical catchment on ferrosols to determine the extent to which reforestation restores key soil properties. Of the nine soil attributes measured (total nitrogen, nitrate and ammonium concentrations, net nitrification and ammonification rates, organic carbon, bulk density, fine root biomass and water infiltration rates), only infiltration rates were significantly lower in pasture than remnant riparian rainforest. Within reforestation plantings, bulk density decreased up to 1.4-fold and infiltration rates increased up to 60-fold with time post-reforestation. Our results suggest that the main outcome of belowground processes of early reforestation is the recovery of the soils' physical structure, with potential beneficial ecosystem services including reduced runoff, erosion and associated sediment and nutrient loads in waterways. We also demonstrate differential impacts of two commonly planted tree species on a subset of soil properties suggesting that preferential planting of select species could accelerate progress on specific restoration objectives.

## Introduction

Globally, riparian zones are widely recognized for their critical role in water regulation [1]–[5], and conservation of biodiversity [6]–[9]. Clearing of vegetation from stream banks can cause increased soil erosion and decreased water quality through the loss of filtration services, in addition to the loss of critical species habitat [10]. In direct response, degraded stream-banks are increasingly being restored to reinstate ecological functions [11]. Riparian zones are also regarded as foci of restoration opportunity as they are often preferentially renounced from agricultural production over agronomically more valuable areas [9].

Soil physical, chemical and functional properties change with succession from pasture to secondary forest including reforestation plantings in the tropics, subtropics and temperate regions [12]–[16]. Soil properties such as bulk density, soil water content, fine root biomass, porosity, stocks of nitrogen, carbon and phosphorus, nitrogen cycling, availability of macro- and micronutrients, as well as toxins (metals, salts), have been studied in context of land use change [17]–[19]. However, most studies focus on mid- to long-term effects of reforestation with less consideration of the short-term effects (<10–20 years) of reforestation plantings, and associated reinstatement of soil functions that might motivate landholders to become actively engaged in restoration. Another knowledge gap is the relative contribution of tree species to outcomes of riparian restoration plantings, and such information could inform decisions on species selection and reforestation goals.

In Australia, riparian reforestation is a major focus of habitat restoration in the tropics and subtropics; over 70% of rainforest restoration projects have targeted banks of rivers and streams [20]. However, much evaluation of the success of reforestation plantings has focused on aboveground attributes [21]–[23], with less regard to belowground processes including soil function (but see Paul [14] for the recovery of soils via different rainforest restoration pathways). Understanding belowground processes is important to quantify the capacity of reforestation to recover ecosystem function.

Soil properties and functions would intuitively be expected to change over time following reforestation towards a state convergent on intact rainforest ecosystems. Studying riparian soil under remnant rainforest, pasture, and within 20 years post reforestation, we addressed three questions (i) how do soil attributes differ between pasture and remnant riparian rainforest, (ii) how do soils change with time following reforestation, and (iii) do tree species affect soils differentially following reforestation?

The study was motivated by the need for restoration of forest cover in water catchments due to the improvements in water quality that can be achieved [24]. The region targeted for our study is located in the Australian subtropics and has experienced large-scale clearing of rainforest since the mid 19^th^ century. The region is an important water catchment that supplies drinking water to more than 100000 people in the region [25], and numerous land owners and stakeholders are committed to ongoing restoration of riparian zones. Our research aimed to generate knowledge of soil properties to direct future reforestation activities.

## Materials and Methods

### 2.1. Study site

This study focused on riparian areas in the Maleny region in southeastern Queensland, Australia (26°45′S, 152°48′E). The climate is classified as subtropical with an annual rainfall of 1900 mm and annual daily mean temperature maximum and minimum of 23.2°C and 14.2°C, respectively. The soils in the region are classified as permeable red clay-loam ferrosols [26], [27] or Nitisol in the US soil taxonomy. Prior to European settlement in the mid-1800s and subsequent land clearing for timber production and agriculture (especially dairy production), the region was dominated by cool-subtropical rainforest classified as complex notophyll vine forest [28], [29].

### 2.2. Sampling design

Field measurements and soil sampling were carried out between May and June 2013. The sampling design was based around riparian reforestation projects established in the region by the Lake Baroon Catchment Care Group (LBCCG). Reforestation sites were grouped into five spatial clusters across the Maleny Plateau (range of elevation 196 to 441 m a.s.l.) to capture broad variation in landform and land use history. A subset of one to three reforestation sites ranging in age between 2 and 20 years were then selected from each cluster for a total of 10 sites (Table 1). Sites were also established in remnant riparian rainforest where available (2 of 5 clusters) and samples were collected from pasture (adjacent to plantings or remnant forest) at all sites in each cluster.

**Table 1:** GPS coordinates and ages of reforestation and remnant sites.

Site type	Age (years)	Site location
Reforestation	3	26°46′02 S, 152°49′38 E
Reforestation	3	26°46′17 S, 152°49′51 E
Reforestation	4	26°45′06 S, 152°50′46 E
Reforestation	6	26°42′01 S, 152°53′58 E
Reforestation	7	26°46′10 S, 152°49′57 E
Reforestation	8	26°44′05 S, 152°50′54 E
Reforestation	11	26°44′46 S, 152°51′13 E
Reforestation	11	26°46′12 S, 152°49′39 E
Reforestation	12	26°46′08 S, 152°49′43 E
Reforestation	20	26°42′01 S, 152°53′58 E
Remnant	-	26°45′07 S, 152°51′02 E
Remnant	-	26°44′50 S, 152°51′11 E

All sites were located on private land in the Maleny region. Pasture sites were located immediately adjacent to each forested site. Ages are based on the year that they were planted relative to 2013.

At each reforestation or remnant site, five subplots were established. Each subplot was deliberately located under a different tree species. The five tree species targeted here were derived from a pool of species commonly used in local rainforest plantings, which in turn were selected to include early to late successional species, nitrogen fixing and/or waterlogging tolerant species. The tree species chosen to investigate species-specific effects on soil properties included *Acacia melanoxylon* R.Br. (Fabaceae, N_2_ fixer, early succession species), *Ficus coronata* Spin (Moraceae, nitrophile, waterlogging tolerant, early succession species), *Flindersia schottiana* F. Muell. (Rutaceae, mid-late succession), *Homalanthus nutans* (G.Forst.) Guill. (Euphorbiaceae, early succession species) and *Podocarpus elatus* R.Br. ex Endl (Podocarpaceae, mid-late succession, gymnosperm). In the event that one of the chosen tree species was absent from a site, a closely related species was substituted or, if not available, subplots were located under a random tree within the site. Subplots were positioned under the canopy of each selected tree (mean distance from stem 89.5 cm, SD 45.2 cm).

At each reforestation or remnant forest site, we performed the following procedures: (1) collected a single 8 cm diameter core to a depth of 30 cm (hereafter “textural core”) and divided it into 0–15 cm and 15–30 cm fractions for textural analysis; (2) performed an infiltration test; and, (3) collected two samples of the surface soil using a 12.5 cm diameter, 8.5 cm tall, 1043 cm^3^ volume bulk density ring (hereafter “bulk density ring”) from each of five subplots for analysis of physical and chemical properties of the soil. In addition, a single texture core and two to four bulk density ring subplots were collected from adjacent pasture and infiltration measurements performed. For the purposes of analysis, samples and infiltration measures from pasture were calculated as means for each cluster.

Overall, we sampled 10 reforestation sites, 2 remnant riparian rainforest sites (Table 1) and 5 pasture clusters comprising 24 cores for soil texture analysis, 150 cores for soil chemical and physical analyses and 24 infiltration tests. All sites were private land holdings and owners approval was obtained by Mark Amos (Lake Baroon Catchment Care Group, Maleny, info@lbccg.org.au). Upon returning from the field, all soil samples were stored at 4 °C in sealed plastic bags prior to respective analyses.

### 2.3. Texture analysis

Changes in soil texture mostly occur due to weathering over very long time periods and as demonstrated by Maloney [13]. Unless there is erosion or deposition of soil material, texture is unlikely to vary over time since reforestation. For this reason, the purpose of conducting a soil texture analysis at each site was not to determine changes due to reforestation, but to characterize and evaluate background variation in soil types among sites that might warrant differential treatments of sites in the analysis. Soil samples were not dispersed prior to analysis and as such, the calculated percentages represent the relative size of secondary particles (aggregates) present rather than primary particle size *sensu stricto*. This was considered a more relevant measure of soil function than particle size determined using standard methods, as it gives a relative measure of soil aggregation and structure. Furthermore, the ferrosols in this study are recognized as being subplastic, with clay-sized particles being primarily composed of sesquioxides that do not readily break down into primary particles [30]. These analyses of soil aggregation were conducted on air-dried soil from 0–15 and 15–30 cm depth cores according to the hydrometer method for soil texture analysis [31], [32]. For each sample, 40 g of air-dried soil was ground and mixed with 100 ml 50 g l^−1^ Sodium hexametaphosphate solution and filled to 500 ml with deionized water. Samples were shaken overnight and then emptied into a 1 l cylinder for analysis. Cylinders were filled to 1 l with deionized water, mixed with a plunger and left to settle. Temperature and specific gravity readings were taken 40 seconds and 7 hours after plunging [32]. Using temperature and specific gravity readings, the fraction of sand, silt and clay sized particles in each soil sample was then calculated as a percentage.

### 2.4. Soil physical properties: bulk density and infiltration rates

Soil bulk density was determined from the second bulk density ring in each subplot. Fresh soil was oven dried to a constant weight at 105 °C. Soil bulk density is expressed as grams of dry soil per volume. To quantify fine root biomass, soil from one bulk density ring per subplot was passed through a sieve (8 mm mesh) and roots were manually removed. Roots were then oven dried at 65 °C to a constant weight, and results expressed as grams of fine roots per kilogram of dry soil.

Infiltration rates were measured using a ponded disk permeameter [33]. Soils were assumed to be at field capacity as the region had received in excess of 30 mm in the week preceding the first set of measurements and over 100 mm in the week prior to the second sampling visit. Infiltration tests were performed at a random, relatively flat area representative of the site. Tests in the adjacent pasture were performed at a random point beyond the farthest extent of the neighbouring forest canopy. Upon setting up of the permeameter, grass and other ground covers were clipped to just above the soil surface and the soil pre-moistened with rainwater to reduce the time needed to reach steady state infiltration. The permeameter was filled with rainwater collected from water tanks on local landholder properties to ensure that water quality was similar to that occurring under natural condition. Once the disk permeameter was in place, measurements of water level changes were recorded until the time interval between measurements was stable. As soils were close to field capacity at the start of measurements, steady state was reached quickly. Infiltration rate was calculated as the average volume change per second and then converted into millimeters per hour.

### 2.5. Soil chemical properties: nitrogen and organic carbon

Total concentrations of soil nitrogen (TN) and organic carbon (SOC) were determined using a TruSpec CHN analyzer (LECO Australia Pty. Ltd., Castle Hill, NSW 2154, Australia), using dried, ground and sieved soil (2 mm sieve). Nitrate (NO_3_^-^) and ammonium (NH_4_^+^) were extracted with potassium chloride (KCl) and quantified via colourimetric assays. To remove NO_3_^-^ and NH_4_^+^ from soil exchange sites, 15 g of fresh sieved soil was mixed with 1 M KCl in a 15 ml falcon tube and shaken on an orbital shaker for 1 hour. Samples were centrifuged at 4000 rpm for 4 minutes and 1 ml of the supernatant was frozen until analysis via colourimetric assays according to standard procedures [34], [35]. Net nitrification and ammonification rates were calculated from the difference in concentration between fresh soil samples and those incubated for 20 days at 28°C. Briefly, 15 g of soil were weighed into 50 ml falcon tubes and incubated for 20 days. Soils were kept at field moisture by watering daily with deionized water. Seeds that germinated during the incubation period were removed. Concentrations of NO_3_^-^ and NH_4_^+^ in the incubated soils following KCl extraction were quantified using colourimetric assays as described above. Net nitrification and ammonification rates are expressed as mg nitrate or ammonium generated per kg dry soil per day.

### 2.6. Statistical analysis

Statistical analyses were performed in R version 3.0.1 [36]. To avoid non-independence among subsamples, we derived the aggregated mean of soil attributes for each site (reforestation or remnant rainforest) or cluster (pasture) and used these values as inputs in all analyses, excepting those determining species-specific effects which used values from a single subplot within each site. Pairwise correlation tests (Spearman's) were performed to examine associations between tested soil attributes. Soil attributes under both pasture and remnant riparian rainforest were directly compared using a one-way analysis of variance (ANOVA). Developmental trajectories of soil attributes within reforested sites (site level and separately for specific tree species within sites) were examined using linear regressions between either age or log-transformed age and individual soil attributes. The age term was log-transformed to account for potential non-linear developmental trajectory of reforestation plantings; often showing a rapid change immediately post planting followed by diminishing returns as the system reaches natural equilibrium. For the purposes of quantifying response trajectories with age, we assume that pasture represents 0 years post reforestation.

## Results

Of the 56 subplots within sites, 47 (84%) were associated with one of the five target trees. *Homalanthus nutans* was the species most commonly absent, being present in 50% of reforested sites. Soil aggregation showed only minor variation among sites and land uses so was not considered further in subsequent analyses (sand sized aggregates: mean = 60%, range = 46–76%, clay sized aggregates: mean = 30%, range = 14–46%).

### 3.1. Correlations between variables

Infiltration rates were inversely correlated with bulk density (P = 0.005) but were not correlated with soil particle size (percentage sand, silt and clay) at either 0–15 cm or 15–30 cm depth (P>0.5, not shown). Bulk density was correlated with both SOC and TN (P = 0.02 and 0.01 respectively) (Table 2). Pairwise correlations revealed strong associations between soil nitrogen-related variables.

**Table 2:** Pairwise correlations (Spearman's) between soil attributes across pasture, remnant rainforest and reforestation sites (n = 5, 2, 10 respectively).

Variable	Variable	Spearman's correlation coefficient	P value
Bulk density	SOC	−0.554	0.023
Bulk density	TN	−0.615	0.010
Infiltration	Bulk density	−0.654	0.005
TN	SOC	0.784	<0.001
Ammonification rate	Nitrification rate	−0.664	0.005
Ammonification rate	Fine root biomass	0.662	0.005
Nitrate	Ammonification rate	−0.529	0.031
Nitrate	TN	0.495	0.045
Nitrate	SOC	0.630	0.008

Only significant pairwise correlations (P<0.05) are shown.

### 3.2. Differences between pasture and remnant rainforest

There was no apparent difference between pasture and remnant riparian rainforest in the tested soil attributes except infiltration rates (Table 3). Infiltration rates were significantly greater in the remnant rainforest (1421±995 mm h^−1^) than in pasture sites (220±151 mm h^−1^) (F = 9.526, DF = 5, P = 0.027, Table 3).

**Table 3:** ANOVA results from a comparison of soil attributes between remnant riparian rainforest (n = 2) and pasture (n = 5).

Variable	ANOVA P value	Mean pasture ±1 SD	Mean remnant ±1 SD	Contrast remnant (R) vs. pasture (P)
**Fine root biomass (g kg^−1^)**	0.09	6.64±4.05	13.00±0.38	NS
**Bulk density (g cm^−3^)**	0.36	0.69±0.08	0.61±0.12	NS
**SOC (%)**	0.60	6.15±1.83	7.31±4.15	NS
**TN (%)**	0.85	0.71±0.14	0.74±0.26	NS
**Nitrate (mg kg^−1^)**	0.26	11.8±7.24	22.7±17.9	NS
**Nitrification rate (mg kg^−1^ soil d^−1^)**	0.65	0.67±0.75	0.39±0.03	NS
**Ammonium (mg kg^−1^)**	0.46	4.36±4.27	7.85±8.10	NS
**Ammonification rate (mg kg^−1^ soil d^−1^)**	0.73	0.65±0.98	0.65±0.98	NS
**Infiltration rate (mm h^−1^)**	**0.03**	**220±151**	**1421±995**	**R>P**

Bold text indicates significance at 0.05 level, NS not significant if P>0.05, SD standard deviation.

### 3.3. Recovery of soil properties under reforestation plantings

**Figure 1:**
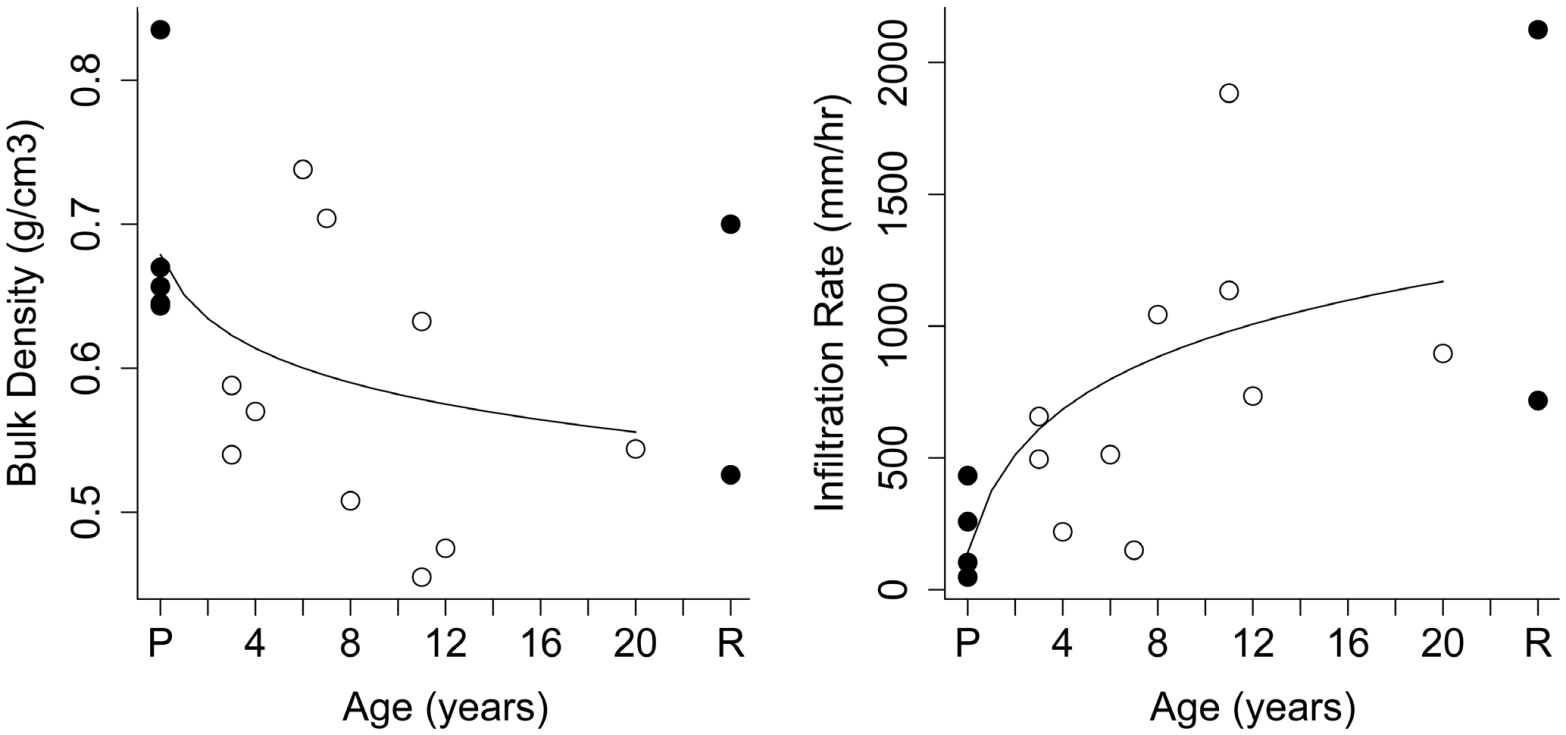
Differences in bulk density (left panel) and infiltration rates (right panel) among pasture (P), reforestation sites aged 2 to 20 years, and remnant rainforest (R) (n = 5, 10 and 2 respectively). Lines show fitted values of the modeled relationship between soil properties and log-transformed age of reforestation. Open circles show reforestation plantings, filled circles indicate pasture clusters and remnant riparian rainforest.

Only bulk density and infiltration rates showed a relationship with age within 20 years post reforestation (Table 4). In each case, models with a log-transformed age term explained more of the variation than models with an un-transformed age term. Bulk density of the soils ranged from 0.312 to 0.991 g cm^-3^, and decreased significantly with time post reforestation (F = 6.312, DF = 13, P = 0.026, Figure 1). Conversely, infiltration rates significantly increased (F = 10.56, DF = 13, P = 0.006) within 20 years post reforestation (Figure 1). Infiltration rates in reforestation sites ranged from 149 mm h^-1^ at a 7-year-old reforestation site to over 1800 mm h^−1^ at an 11-year-old reforestation site. Infiltration rates of pasture sites varied from 3 mm h^−1^ to over 650 mm h^−1^, although this variation was slightly less when data was aggregated by cluster.

**Table 4:** Relationship between tested soil attributes and the age of the reforestation sites (n = 15).

Variable	Age				Log Age			
	Slope	R^2^ value	F statistic	P value	Slope	R^2^ value	F statistic	P value
Fine root biomass (g kg^−1^ soil)	0.02	0.001	0.02	0.90	1.14	0.03	0.34	0.57
Bulk density (g cm^−3^)	−0.009	0.28	5.16	**0.04**	−0.12	0.32	6.31	**0.03**
SOC (%)	−0.03	0.01	0.15	0.70	−0.03	<0.001	0.002	0.97
TN (%)	−0.001	0.004	0.05	0.83	0.005	<0.001	0.004	0.95
Nitrate (mg kg^−1^)	0.09	0.002	0.03	0.86	3.58	0.03	0.38	0.55
Nitrification rate (mg kg^−1^ d^−1^)	0.03	0.13	1.93	0.19	0.30	0.07	0.95	0.35
Ammonium (mg kg^−1^)	−0.09	0.04	0.51	0.49	−1.72	0.11	1.55	0.24
Ammonification rate (mg kg^−1^ d^−1^)	−0.03	0.11	1.54	0.24	−0.47	0.15	2.25	0.16
Infiltration rate (mm h^−1^)	54.9	0.39	9.77	**0.008**	678	0.45	10.6	**0.006**

Pasture is assumed to be 0 years post reforestation. Results from linear models with both age and log-transformed age are shown. P values in bold are significant at the 0.05 level.

### 3.4. Species specific effects on the recovery of soils under reforestation plantings

**Figure 2:**
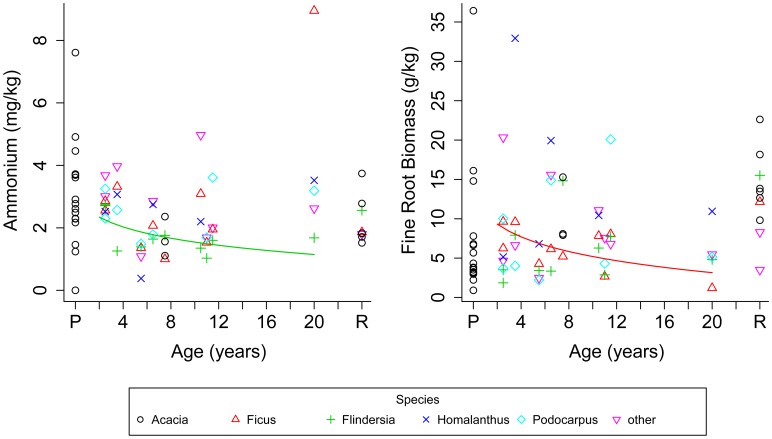
Differences in ammonium (left panel) and fine root biomass (right panel) over time under five tree species in pasture (P), reforestation sites aged 2 to 20 years, and remnant rainforest (R). Genus names refer to the selected tree species under which the soil samples were taken. “Other” refers to soil samples taken from non-specified tree species or pastoral sites. Lines show fitted values of the modeled relationship between individual soil properties and log-transformed age for species with significant models - *Flindersia schottiana* (a) and *Ficus coronata* (b).

Species-specific effects were apparent for two of five tree species when tested soil attributes were modeled against log-transformed age (Figure 2). Ammonium concentrations in soil associated with *Flindersia schottiana* decreased with time post reforestation (F = 4.97, DF = 8, P = 0.056). Fine root biomass in soil associated with *Ficus coronata* decreased through time within 20 years post reforestation (F = 4.921, DF = 8, P = 0.057).

## Discussion

Replanting riparian areas can be driven by a variety of objectives including increasing landscape connectivity for wildlife movements and biogeochemical functions such as trapping nutrients, sediment, pesticides, bank stabilisation, improved water quality and flood mitigation [10]. Here, we studied the trajectory of riparian reforestation to identify key changes in soil attributes through the transition from pasture to riparian tree plantings of increasing age and remnant rainforest. We showed that the major belowground outcomes within 3 to 20 years since planting are physical and associated functional attributes of the soil.

### 4.1. Differences in soil properties between pasture and remnant rainforest

Infiltration rate was the only soil variable studied here that differed significantly between pasture and remnant riparian rainforest. Pasture infiltration rates were ≈15% of those of riparian rainforest, consistent with the finding of an infiltration rate in pasture 20% of that in remnant rainforest by Peñuela and Drew [15]. We detected no statistically significant differences in other soil properties between pasture and remnant rainforest soils. Limited effects of land use on selected chemical properties are consistent with previous findings in Australian on ferrosol soils [14]. However, it was expected that attributes such as bulk density and fine roots would differ between land use types [14]. It is possible that these attributes differ at a scale inscrutable beyond background variability between sites, possibly necessitating longitudinal study of permanent plots following reforestation. Limited availability of remnant forest within sampling clusters, a consequence of extensive clearing of riparian rainforest in the region, was also a contributing factor in the comparatively low statistical power for detecting changes in measured attributes. Nevertheless, the data also indicate that there is little degradation of soil with respect to N and C stock in the studied systems.

### 4.2. Recovery of soils under reforestation plantings

Bulk density and infiltration rates both changed significantly with time under the reforestation plantings. Bulk density showed a negative trend with age whereas infiltration increased with time in the reforestation plantings. The observed trend for decreasing bulk density is consistent with previous studies globally [9], [14], [37], [38]. The higher bulk density and lower infiltration rates of the pastures may be partially attributed to compaction by livestock and/or machinery [39]. Lower bulk densities are often associated with soil of forests and reforestation plantings because greater presence of roots and soil biota and higher concentrations of organic matter enhance soil porosity. In remnant forest and reforestation plantings, the high turnover of roots results in more open soil pores as roots grow and subsequently die [40]. Additionally, pastures generally have more shallow and non-woody roots while forests have a broader range of fine to large woody roots including with larger, woody roots that leave conduits in the soil post death [41]–[43].

Higher densities and diversities of soil organisms in forests and reforestation plantings [44], [45] contribute to the aeration of soil and reduce bulk density. High soil organic matter is also associated with low bulk density [18], a trend seen in this study as bulk density was negatively correlated with SOC. This was expected as SOC is indicative of litter dynamics, turnover and decomposition rates and is responsible for providing low-density mass to the soil and improving soil structure [15], [18], [37], [46]. The bulk density values in our study (0.65 g cm^−3^) are low in comparison to average soils (*>*1.2 g cm^−3^). Paul [14] also reported low bulk densities (0.6–0.8 g cm^−3^) for similar ferrosols in reforestation plantings and remnant rainforest, confirming the low bulk density of this soil type. We attribute these low bulk densities to the friable nature of the ferrosols in the study region, high porosity and the high organic matter content in the topsoil. We also propose that the low bulk density contributes to the trend of increasing infiltration rates with time following reforestation as the two variables were inversely correlated.

Similar to prior studies, we found a significant increase in infiltration rates with reforestation age, with infiltration rates of the pastures being always lower than those of adjacent reforestation sites. We note a strong positive pattern with age within the first 5–10 years post reforestation, indicating that even young tree plantings are able to promote increased infiltration rates [37], [47]. The trajectory in infiltration rates from the plantings (up to 20 years old) indicate that increases of 182 mm h^−1^ can be expected for each year in the first decade since planting, with most gains seen within the first 10 years post-reforestation. This trend is consistent with findings of Bharati [48] of short term changes in infiltration rates under riparian buffers when compared to adjacent cultivated fields and pastures, noting a 5-fold increase in infiltration rates under riparian buffers after 6 growing seasons. Our finding that infiltration rates are strongly enhanced soon after reforestation has meaningful consequences for decision making on land use, water quality and conservation with reduced run-off, slowed surface erosion and reduced non-point source pollution as beneficial outcomes [49].

Here, two of the tested nine soil attributes varied between pasture and reforestation sites. One possible explanation is the recentness of the plantings. Reforestation sites were relatively recent compared to studies that focused on plantings of much greater age (up to 60 years) [13], [38], [43] and this may have contributed to the uniformity between sites in our study. However, the common lack of differences between the studied pasture and remnant riparian rainforest sites suggests that inherent high soil fertility may be a larger contributor to soil properties than land cover, as has been suggested previously [43], [50].

### 4.3. Species specific effects on the recovery of soils under reforestation plantings

In previous studies, species-specific effects were not used to help elucidate changes in soil properties post-reforestation [14], [51], although it is known that species-specific effects can modify soil properties [52]. We detected two species-specific effects in the five tree species and eight variables examined. *Ficus coronata* and *Flindersia schottiana* showed negative relationships with time since reforestation for fine root biomass and soil ammonium levels respectively. We hypothesized that as reforested sites become more established, fine root biomass increases to access nutrients in the surface soil layer of rainforests [41], [43], [53]. The studied *F. coronata* specimens displayed the opposite trend to that expected, showing a negative fine root relationship with time post-reforestation. This decreasing trend exhibited by *F. coronata* may be explained by a need for greater structural support through large woody roots in which they invest carbon at the expense of fine surface roots, or the ability to access nutrients from the deeper soil, however greater sampling at depth would be needed to confirm these hypotheses. Nevertheless, if verified, an ability to shift root production into in deeper layers could be a useful trait providing physical impediment in the subsoil, particularly where greater soil stability is a desired goal of reforestation.

Another species-specific effect was observed with *F. schottiana* showing a decreasing relationship with ammonium concentrations and time post-reforestation. This result was not entirely unusual as ammonium is readily converted to nitrate in some soils and nitrate may be preferentially used by plants [54]. However, we did not detect higher nitrate concentration in soil under *F. schottiana*, which would be expected if nitrification is simulated. This may be due to high N uptake and demand of *F. schottiana,* which was not investigated here. However, this finding conforms broadly to the results of prior studies [14], [38], [55], which saw that ammonium levels were highest in pasture and lowest in forest.

It is noteworthy that the species-specific effects detected were not the same as those that were seen at a site level. When means at the site level were considered, only infiltration and bulk density were significant, however at a species level, both *F. schottiana* and *F. coronata* showed significant variations through time for ammonium levels and fine root biomass respectively. This suggests that while individual species may be able to preferentially alter their immediate surroundings, their influence is diminished at a site level. A possible explanation is that the diverse mix of native tree species, representing a variety of physiotypes, and the individual species-specific effects on soil are less influential than the effects of the overall plantings on soil properties. Had the reforestation planting been of a simpler species mix or the relative proportion of select species been greater, soil traits may have reflected more strongly particular species-specific effects. This indicates that particular reforestation outcomes may be facilitated by explicit selection of certain tree species. Further studies have to determine the efficacy and benefits of a less species-rich reforestation species mixture.

## Conclusion

Reduced bulk density and enhanced infiltration rates were the key immediate outcomes resulting from riparian reforestation plantings. It is noteworthy that the most change occurred within just 5 to 10 years post-planting suggesting that this form of active restoration can reinstate important soil functions within a comparatively short time. Given the linkages between increased infiltration and ecosystem services such as nutrient buffering, filtration of runoff and sediments, we conclude that the reforestation is, by extension, achieving the objective of improving water quality. This finding has consequences for land managers and conservationists interested in lowering runoff, non-point source erosion and limiting loss of topsoil, all of which are major concerns and motivators for reforestation. Further, soil infiltration rates are intricately linked with several soil properties influenced by slight changes in soil structure well before changes are perceivable in other soil properties [56]. This signifies that the reforestation plantings are on a positive trajectory to restoring other soil properties and ecosystem function with infiltration rates as front-runner in these changes.

## Supporting Information

Table S1 (XLSX)
